# Diagnostic value of plasma HSP90α levels for detection of hepatocellular carcinoma

**DOI:** 10.1186/s12885-019-6489-0

**Published:** 2020-01-02

**Authors:** Wene Wei, Mengshu Liu, Shufang Ning, Jing Wei, Jianhong Zhong, Jilin Li, Zhengmin Cai, Litu Zhang

**Affiliations:** 1 Department of Research, Affiliated Tumor Hospital of Guangxi Medical University, Nanning, 530021 Guangxi Zhuang Autonomous Region China; 2 Department of Hepatological Surgery, Affiliated Tumor Hospital of Guangxi Medical University, Nanning, 530021 Guangxi Zhuang Autonomous Region China

**Keywords:** Hepatocellular carcinoma, Biomarker, Plasma HSP90α, Diagnostic value

## Abstract

**Background:**

Hepatocellular carcinoma (HCC) is a major health problem worldwide. However, the popular tumor marker, AFP, lacks sensitivity although its specificity is high. Tissue biopsy is an invasive operation and may increase the risk of needle-track metastases. Heat shock protein 90 (HSP90) is a potential biomarker for tumor diagnosis and prognosis. This study aims to determine whether levels of plasma HSP90α in HCC patients can be used as a cost-effective and simple test for the initial diagnosis of the disease.

**Methods:**

Plasma samples were collected from 659 HCC patients, 114 secondary hepatic carcinoma (SHC) patients, 28 hepatic hemangioma patients and 230 healthy donors. The levels of HSP90α were measured by ELISA.

**Results:**

The levels of plasma HSP90α in HCC patients were significantly higher than in healthy donors and in patients with hepatic hemangioma or SHC (144.08 ± 4.98, 46.81 ± 1.11, 61.56 ± 8.20 and 111.96 ± 10.08 ng/mL, respectively; *p* < 0.05 in all cases). The levels were associated with age (*p* = 0.001), BCLC stage (*p* < 0.001), levels of AFP (*p* < 0.001), tumor size (*p* < 0.001), tumor number (*p* < 0.001), PVTT (*p* < 0.001), EHM (*p* < 0.001) and Child-Pugh stage in the HCC cohort. In addition, the levels of plasma HSP90α showed an upward trend along with the progression of the BCLC stage. ROC curve analysis showed that compared to AFP (AUC 0.922, 95%CI 0.902–0.938) or HSP90α (AUC 0.836, 95%CI 0.810–0.860), the combination of HSP90α and AFP (AUC0.943, 95%CI 0.925–0.957) significantly improved the diagnostic efficiency for HCC patients.

**Conclusion:**

The results suggest that plasma Hsp90 α levels can be used as an initial diagnosis for patients with HCC in both rural and cosmopolitan settings.

## Background

Liver cancer is composed mostly of primary liver cancers and secondary liver cancers. Hepatocellular carcinoma (HCC), as the major type of primary liver cancer, is the fifth most common tumor worldwide and the third leading cause of cancer mortality, responsible for 745,500 cancer deaths annually [1]. Moreover, more than 50% of HCC-related deaths occurred in China [2]. In spite of great progress for HCC therapy in recent years, the prognosis remains poor, due to the high incidence of recurrence and metastasis and the 5-year-survival rate has remained at less than 12% [3]. To the best of our knowledge, most of HCC patients are diagnosed at an advanced stage due to lack of typical clinical manifestations and awareness of disease screening. Nowadays, the HCC screening is based on measurement of serum alpha-fetoprotein (AFP) as well as imaging technologies and histology [4, 5]. However, the conventional liver imaging for HCC does not perform well on tumors of less than 1 cm and AFP lacks adequate sensitivity and specificity in patients with atypical AFP levels. In addition, although tissue biopsies can make an accurate judgment, it is an invasive operation and may increase the needle-track metastases [6, 7]. Therefore, non-invasive and more effective biomarkers for HCC are urgently needed.

Heat shock protein 90 (HSP90) is an evolutionarily highly conserved intracellular molecular chaperone that is usually induced in response to cellular stress. It assists the maturation of an array of client proteins. The HSP90 family is composed of four major members: HSP90α, HSP90β, Grp94 and TRAP1. HSP90α and HSP90β are located mainly in the cytoplasm, and the other two proteins are located mainly in the endoplasmic reticulum and mitochondrial matrix, respectively. Due to its key roles in modulating signal transduction, especially in tumor cells, HSP90α has become a research hotspot. A large sample data study has shown that the plasma levels of HSP90α in lung cancer patients are significantly higher compared to healthy controls [8]. In addition, a recent study showed that plasma HSP90α can discriminate patients with liver cancer from non-liver cancer controls [9]. However, the plasma HSP90α levels were not elevated in benign liver tumors and secondary hepatic carcinoma (SHC) patients in this study. In order to verify the presence of a stable and reliable biomarker, a large amount of data and studies are required for verification. Therefore, in order to establish whether plasma HSP90α levels can be used as a biomarker for HCC in the clinic, in the current study we measured plasma HSP90α levels in HCC and SHC patients as well as benign liver tumor cohort.

## Methods

### Patients

From January 1, 2018 to February 28, 2019, a total of 801 liver disease patients in the Hepatobiliary Surgery Department of the Affiliated Tumor Hospital of Guangxi Medical University were enrolled in this study. The subjects included were 659 HCC patients, 114 SHC patients and 28 patients with hepatic hemangioma (HH). HCC was diagnosed according to the American Association for the Study of Liver Diseases (AASLD) guidelines. All patients were scanned by means of magnetic resonance imaging, abdominal B ultrasound and computed tomography, and were examined for clinical symptoms and signs of disease, together with measurement of serum AFP levels. In addition, none of the patients were subjected to any anti-tumor treatment or surgical resection at the time of diagnosis. The clinical features were obtained from the electronic records. Tumor stage was determined according to the Barcelona Clinic Liver Cancer (BCLC) staging system. The control group included 230 healthy donors (HD). All controls and patients provided written informed consent. This study was approved by the Local Ethics Committee of the Affiliated Tumor Hospital of Guangxi Medical University, and it was conducted in accordance with the Declaration of Helsinki and current hospital ethical guidelines.

### Assessment of HSP90α and AFP levels

The levels of plasma HSP90α were measured by using the ELISA kit for HSP90α protein (Yantai Protgen Biotechnology Development Co., Ltd., Yantai, China). 2 mL of fresh blood samples with EDTA-K2 anticoagulant were collected from patients and controls. All the samples were collected prior to anti-cancer treatment or surgery. All the operations were followed according to the manufacturer’s instruction. The kits were first pre-incubated at 37 °C for 30 min. The samples were prepared for ELISA analysis: a. the fresh blood samples were centrifuged at 3000 rpm for 10 min; b. the plasma was removed and diluted 20 times with the diluent solution provided. Then, the standards were loaded together with the quality controls and the prepared samples (50uL of each) were added into 96-well plates followed by addition of 50uL of anti–Hsp90aHRP-conjugated antibody. These were incubated at 37 °C for 1 h after gentle shaking. Then, the plates were washed six times with the wash buffer provided which was proceeded by the chromogenic reaction; 50uL peroxide and 50uL 3, 3′, 5, 5′ -tetramethylbenzidine and incubation at 37 °C for 20 min and the reaction was terminated by addition of an acid stop buffer. Finally, the optical density was measured by using a spectrophotometer at 450 nm for the detection wavelength with 620 nm as the reference wavelength. The concentration of HSP90α protein in each sample was calculated according to a standard curve of optical density values. The levels of serum AFP were measured using electro-chemiluminescence immunoassay kits (Cobas, Roche Diagnostics, Germany) according to the manufacturer’s instruction. Serum samples were obtained in a similar way to those of plasma, but blood samples were initially placed in tubes without anticoagulant and treated as described above.

### Statistical analysis

All quantitative data are presented as the mean ± SE. The HH patients were analyzed as the benign liver tumor group. The one-way ANOVA was performed using SPSS 17.0 software (SPSS, Chicago, IL, USA). The scatter plots were performed using GraphPad Prism 7 software (GraphPad Software, Inc., San Diego, CA, USA). The paired comparison of ROC curves was performed using MedCalc version 18.11.3. The optimum cut-off value was determined by using the quantity corresponding to the maximum value of the Youden’s index (Youden’s index = sensitivity + specificity - 1) [10].

## Results

A total of 901 cases in this study consisted of 659 HCC patients, 114 SHC patients, 28 HH patients and 230 healthy controls (Raw data for each cohort are atttached in Additional files 1, 2, 3 and 4). The median ages in each group were 51, 61, 47 and 37 years, respectively.

### Comparison of HSP90α levels between groups

**Fig. 1:**
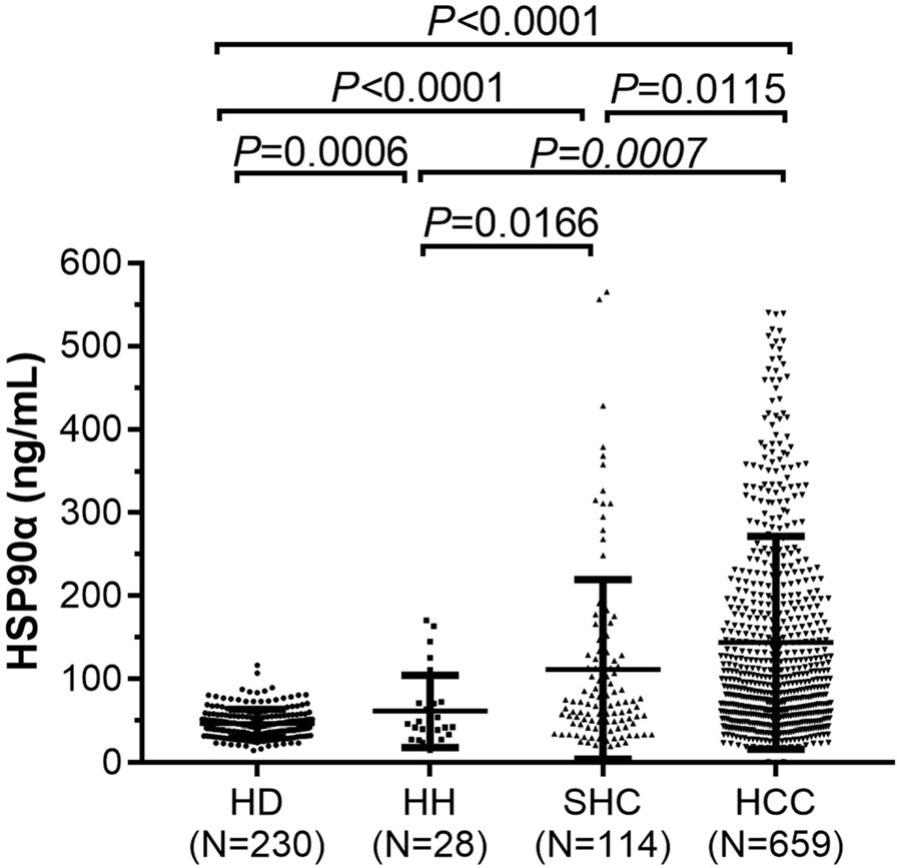
Levels of plasma HSP90α in the different groups

The plasma levels of HSP90α in the different groups of patients and controls are shown in Fig. 1. The levels of plasma HSP90α in HD, HH, SHC and HCC cohorts were 46.81 ± 1.11, 61.56 ± 8.20, 111.96 ± 10.08 and 144.08 ± 4.98 ng/mL, respectively. Statistical analysis showed that HSP90α was at significantly higher levels in HH, SHC and HCC patient cohorts when compared to the HD cohort (*p* < 0.001, *p* < 0.001, *p* < 0.001, respectively). In pairwise comparisons, the plasma HSP90α showed significantly higher levels in HCC patients when compared to the HH and SHC patients groups (*p* < 0.001 and *p* = 0.011, respectively).

### Associations between HSP90α levels and clinical characteristics of HCC patients

**Fig. 2:**
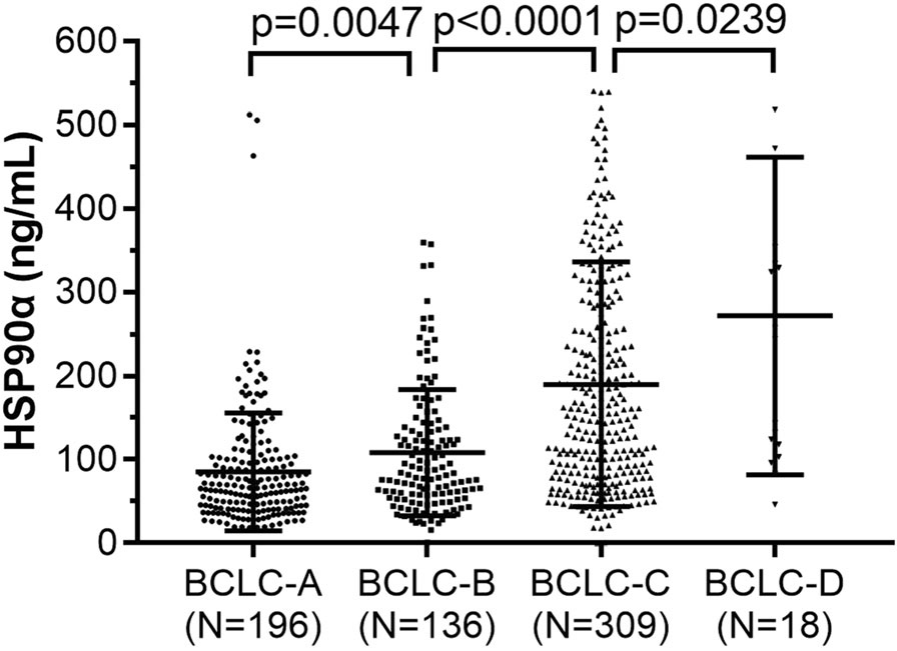
Levels of plasma HSP90α and pairwise comparison in BCLC staging system groups

The relationship between the HSP90α levels and clinical characteristics in HCC patients are shown in Table 1. The levels of HSP90α showed no statistical difference with gender, liver cirrhosis and HBV infection status (*p* = 0.419, *p* = 0.099 and *p* = 0.605, respectively). Moreover, what is noteworthy is that the plasma HSP90α showed a remarkable higher level in a younger group of patients compared with an older age group (162.56 ± 8.22 vs. 129.02 ± 5.96 ng/mL, *p* = 0.001). With respect to the BCLC stage, the levels of the HSP90α in BCLC-A, BCLC-B, BCLC-C and BCLC-D stage were 85.07 ± 5.03, 108.11 ± 6.47, 189.91 ± 8.33 and 271.84 ± 44.75 ng/mL, respectively. In addition, the data showed that the plasma HSP90α levels were different at the BCLC stage sub-group (*p* < 0.001) and a remarkable upward trend with the progress of BCLC stage (Fig.2, *p* < 0.05 in all paired-comparisons). Also, the levels of plasma HSP90α in HCC patients with a greater than or equal to an AFP concentration of 400 ng/mL were higher than patients with an AFP of less than 400 ng/mL (182.10 ± 8.41 vs. 110.93 ± 5.14 ng/mL, respectively, *p* < 0.001). Moreover, the levels of plasma HSP90α showed a significantly higher difference in HCC patients with a larger size of tumor (≥5 cm) as well as those with multiple tumors (all *p* < 0.001). The presence of portal vein tumor thrombus (PVTT) or extrahepatic metastasis (EHM) was also associated with high levels of plasma HSP90α (113.58 ± 5.21 vs. 196.65 ± 9.24 ng/mL, 127.49 ± 5.03 vs. 220.97 ± 13.55 ng/mL, respectively, all *p* < 0.001). Furthermore, the HCC patients with poor Child-Pugh B/C showed significantly higher levels of plasma HSP90α compared to the HCC patients with Child-Pugh A (*p* < 0.001).

**Table 1:** Associations between plasma HSP90α levels and clinical characteristics of HCC patients

Clinico-pathological characteristics		N (659)	HSP90α (ng/mL)	*P*
Age (years)	< 50	296	162.56 ± 8.22	0.001
	≥50	363	129.02 ± 5.96	
Gender	Male	568	145.70 ± 5.45	0.419
	Female	91	134.02 ± 11.91	
BCLC stage	A	196	85.07 ± 5.03	0.000
	B	136	108.11 ± 6.47	
	C	309	189.91 ± 8.33	
	D	18	271.84 ± 44.75	
AFP (ng/mL)	< 400	352	110.93 ± 5.14	0.000
	≥400	307	182.10 ± 8.41	
Liver Cirrhosis	Absent	367	151.42 ± 7.10	0.099
	Present	292	134.86 ± 6.80	
HBV infection	Absent	178	139.84 ± 8.81	0.605
	Present	481	145.65 ± 6.00	
Tumor size	≥5 cm	483	168.09 ± 6.21	0.000
	2–4.99 cm	146	78.72 ± 5.40	
	< 2 cm	30	75.69 ± 11.37	
Tumor number	Single	363	124.66 ± 5.63	0.000
	Multiple	296	167.92 ± 8.48	
PVTT	Absent	417	113.58 ± 5.21	0.000
	Present	242	196.65 ± 9.24	
EHM	Absent	542	127.49 ± 5.03	0.000
	Present	117	220.97 ± 13.55	
Child-Pugh class	A	466	121.40 ± 5.23	0.000
	B	164	200.40 ± 11.49	
	C	29	190.14 ± 24.27	

*BCLC* Barcelona Clinical Liver Cancer; *AFP* alpha-fetoprotein; *HBV* hepatitis B virus; *PVTT* portal vein tumor thrombus; EHM extrahepatic metastasis

### The diagnostic efficiency of HSP90α and AFP for determination of hepatic malignancy

**Fig. 3:**
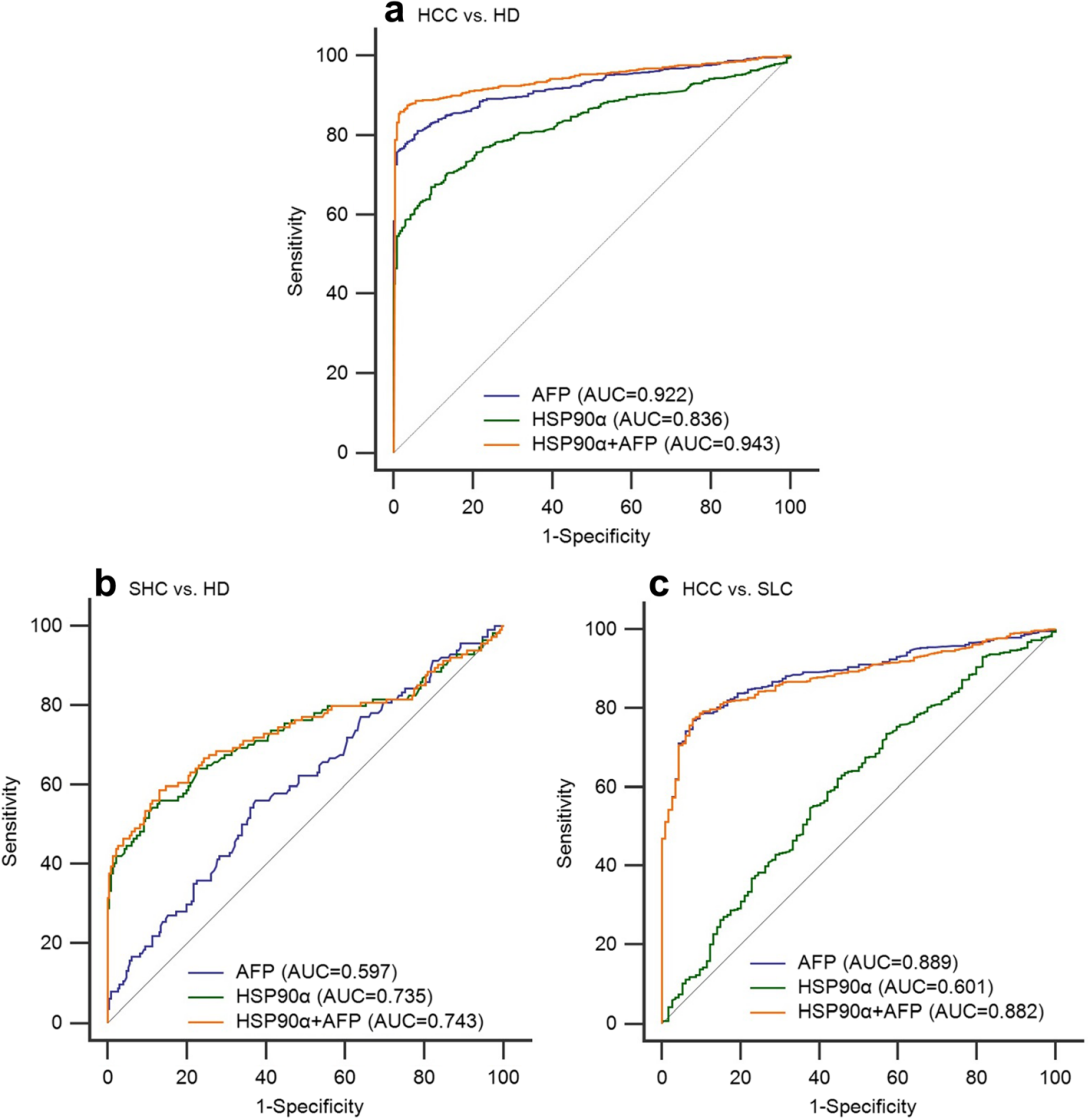
The ROC curve analysis the diagnosis efficency of HSP90α and AFP for HCC and SHC. **a** The diagnostic ability to distinguish HCC patients from healthy donors. **b** The diagnostic ability to distinguish SHC patients from healthy donors. **c** The diagnostic ability to distinguish HCC patients from those with SHC

The ROC curve analysis was conducted to assess the diagnostic efficiency of HSP90α and AFP in determining hepatic malignancy and the results are shown in Fig. 3. The analysis of hepatic malignancy was performed after dividing the patients into two groups: an HCC and a SHC cohort.

**Fig. 4:**
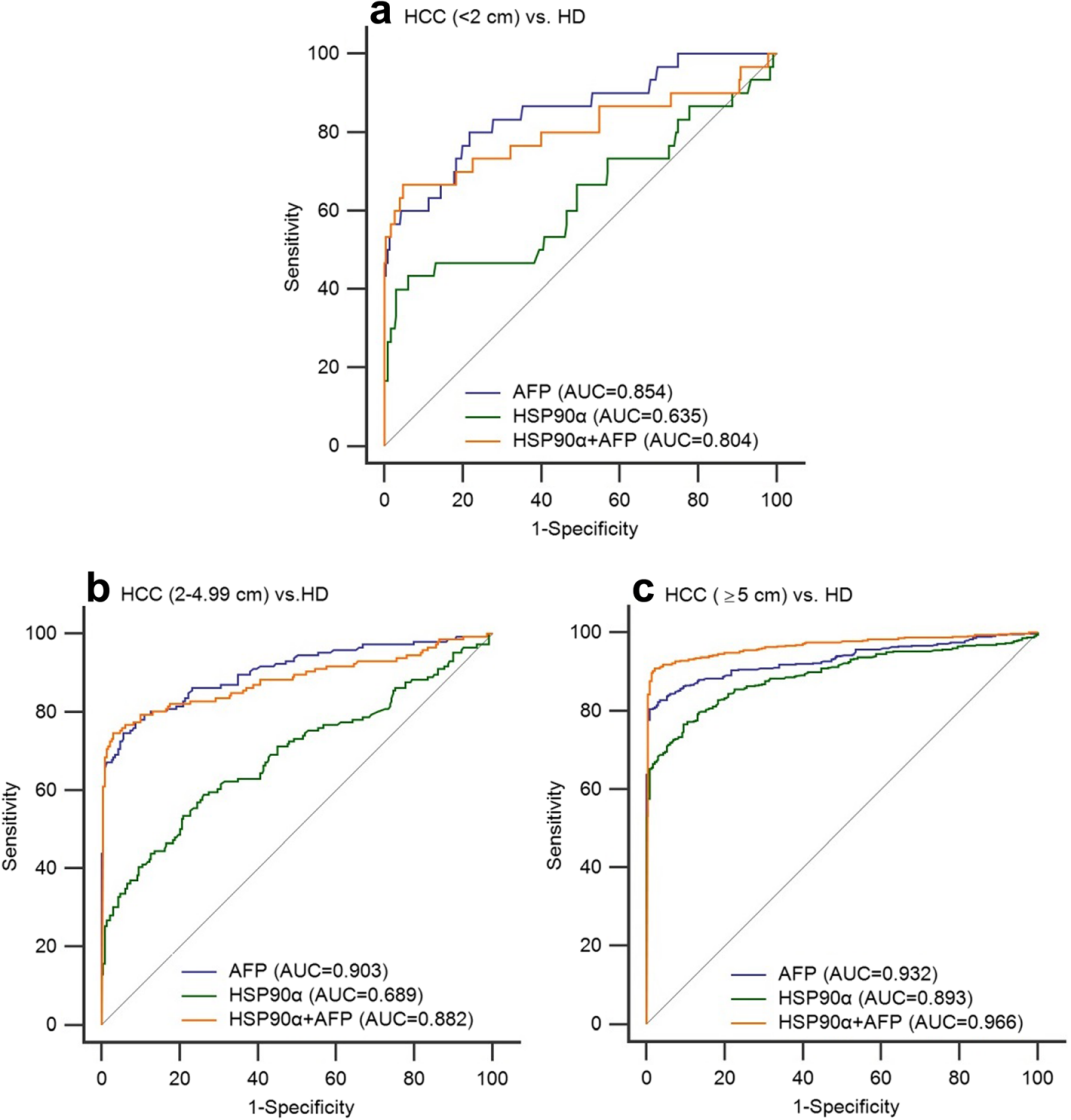
The ROC curve analysis the diagnosis efficency of HSP90α and AFP for tumor size in HCC patients. **a** The diagnostic ability to distinguish HCC patients with tumor size less than 2 cm from healthy donors. **b** The diagnostic ability to distinguish HCC patients with tumor size 2–4.99 cm from healthy donors. **c** The diagnostic ability to distinguish HCC patients with tumor size greater than or equal to 5 cm from healthy donors

**Fig. 5:**
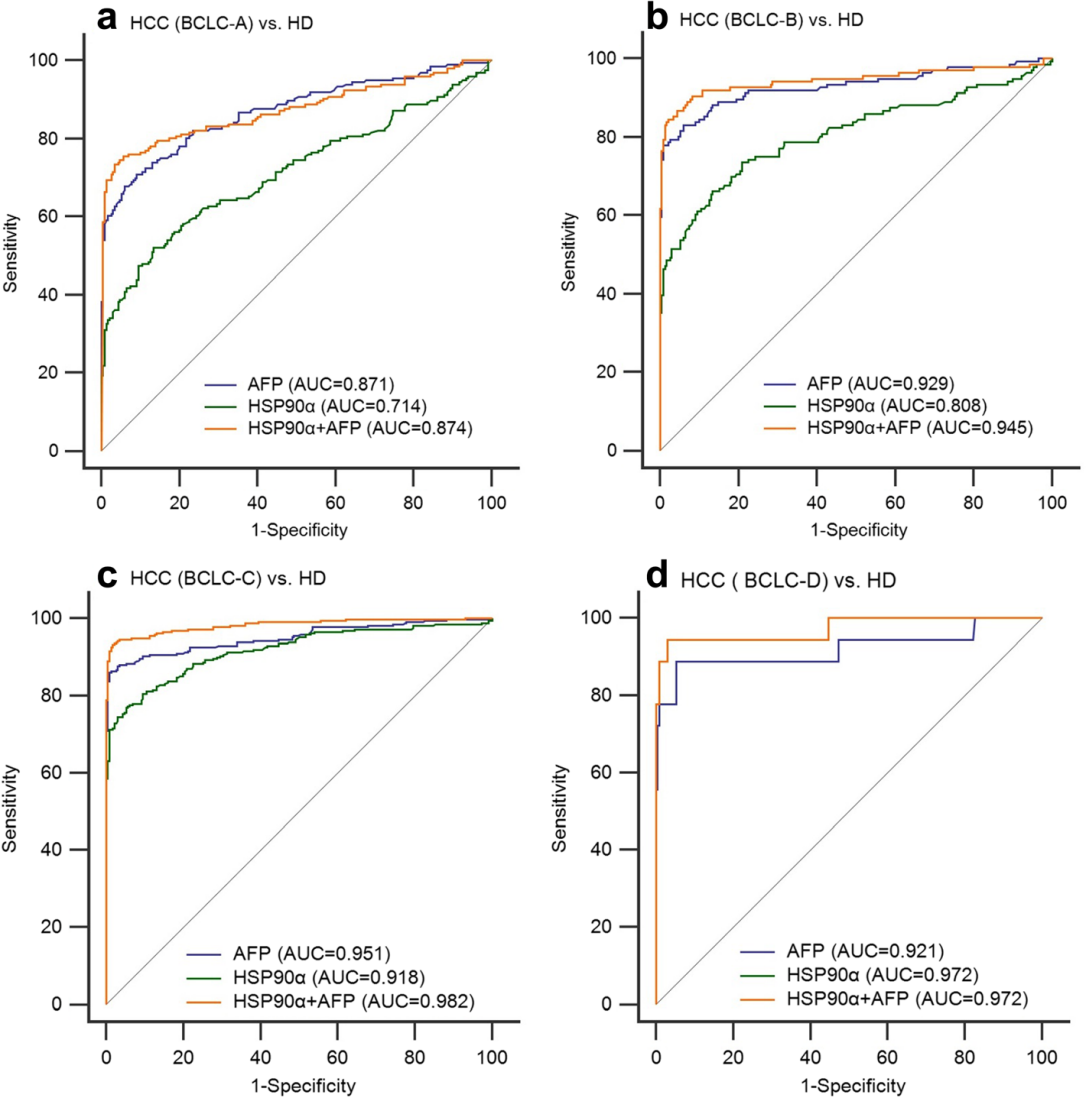
The ROC curve analysis the diagnosis efficency of HSP90α and AFP for BCLC stage in HCC patients. **a** The diagnostic ability to distinguish HCC patients with BCLC-A stage from healthy donors. **b** The diagnostic ability to distinguish HCC patients with BCLC-B stage from healthy donors. **c** The diagnostic ability to distinguish HCC patients with BCLC-C stage from healthy donors. **d** The diagnostic ability to distinguish HCC patients with BCLC-D stage from healthy donors

The diagnostic efficiency of HSP90α and AFP showed a better performance in the HCC cohort (AUC 0.836, sensitivity 67.07%, specificity 90.43%; AUC 0.922, sensitivity 81.18%, specificity 93.91%; respectively, Fig. 3a, Table 2) than in the SHC cohort (AUC 0.735, sensitivity 56.14%, specificity 86.96%; AUC 0.597, sensitivity 56.14%, specificity 62.61%, respectively; Fig. 3b, Table 2) when compared to healthy donors. In addition, the combination of HSP90α and AFP significantly improved the diagnostic ability of HCC from healthy donors (AUC 0.943, sensitivity 85.89%, specificity 98.26%, Fig.3a, Table 2). However, when we focus on the diagnostic ability of HCC from SHC, the serum AFP (AUC = 0.889, sensitivity 76.9%, specificity 92.1%) was better than plasma HSP90α (AUC = 0.601, sensitivity 63%, specificity 54.4%) for distinguishing the HCC patients from those with SHC (Fig. 3c). Subsequently a subgroup analysis was conducted to evaluate the plasma HSP90α initial diagnosis value for early HCC patients and the results demonstrated that plasma HSP90α had a poor performance for the initial diagnosis of early HCC when patients had tumors of less than 2 cm (AUC = 0.635, Fig. 4a) or the early stage of HCC as characterized by patients at BCLC-A stage (AUC = 0.714, Fig. 5a).

**Table 2:** Main parameters of ROC curve analysis results and the pairwise comparison of the ROC curves

Variable	AUC	95%CI	Sensitivity (%)	Specificity (%)	Cut-off	*p*
HCC-HD						
AFP	0.922	0.902–0.938	81.18	93.91	5.38	< 0.001
HSP90α	0.836	0.810–0.860	67.07	90.43	69.10	< 0.001
AFP+ HSP90α	0.943	0.925–0.957	85.89	98.26		< 0.001
Pairwise comparison						
AFP ~ HSP90α		0.057–0.115				< 0.001
AFP ~ AFP + HSP90α		0.006–0.036				< 0.005
HSP90α ~AFP + HSP90α		0.085–0.128				< 0.001
SHC-HD						
AFP	0.597	0.543–0.649	56.14	62.61	2.68	0.003
HSP90α	0.735	0.685–0.781	56.14	86.96	64.70	< 0.001
AFP+ HSP90α Pairwise comparison	0.743	0.693–0.788	58.77	86.96		< 0.001
AFP ~ HSP90α		0.054–0.223				0.001
AFP~AFP + HSP90α		0.064–0.228				< 0.001
HSP90α~AFP + HSP90α		−0.004-0.012				0.203
HCC-SHC						
AFP	0.889	0.864–0.910	76.9	92.10	7.83	< 0.001
HSP90α	0.601	0.566–0.636	63.00	54.40	75.45	0.001
AFP+ HSP90α Pairwise comparison	0.882	0.857–0.903	77.24	92.11		< 0.001
AFP ~ HSP90α		0.227–0.348				< 0.001
AFP~AFP + HSP90α		−0.008-0.022				0.341
HSP90α ~AFP + HSP90α		0.210–0.350				< 0.001

*HD* healthy donors; *HH* hepatic hemangioma patients; *SHC* secondary hepatic carcinoma patients; *HCC* hepatocellular carcinoma patients; *AFP* alpha-fetoprotein

## Discussion

HCC is a major health problem worldwide, with more than 700, 000 cases diagnosed annually and with a 1-year survival rate of 47%, and a 5-year survival rate of 10% [1, 11]. The decrease in survival rate after the first year is highly significant. Although risk factors (such as cirrhosis of the liver) are recognized, they are the third leading cause of tumor-related mortality. Since there are no obvious symptoms at the early stages, there are still huge challenges in the early diagnosis of high-risk groups. The biomarker, AFP, has been used widely over the last 40 years. However, its sensitivity and specificity is limited for diagnosis of HCC [12]. The identification of new tumor biomarkers could be pivotal for the improvement of patient diagnosis and survival.

HSP90α, is an abundant intracellular chaperone and it has been shown to be located in the extracellular space [13, 14]. Moreover, increasing evidence have demonstrated that HSP90α is widely recognized to have a role in modulating the conformation, stability and function of oncogenic proteins, and that it is involved in cell proliferation, apoptosis, cell cycle progression, migration and invasion [15–20]. In addition, previous studies have shown that the high levels of protein HSP90α are associated with the occurrence of solid malignant tumors [9, 19, 21].

In the present study, the levels of plasma HSP90α were significantly higher in hepatic malignancy compared to a healthy donor cohort or patients with benign liver tumors. This finding was in accordance with previous studies [9, 19]. Beyond that, the difference is that in this study, plasma HSP90α levels were assessed in secondary hepatic carcinoma cohorts, ant the results showed that the plasma HSP90α levels were also significantly higher in both an HCC cohort and a SHC cohort compared to patients with hemangioma, respectively. In addition, plasma HSP90α levels were significantly higher in the HCC cohort compared to the SHC cohort. Therefore we speculate that the levels of plasma HSP90α might be a potential cancer-specific biomarker for diagnosis of hepatic malignancy and have the ability to distinguish between primary and secondary hepatic cancer. The purpose of this study was to investigate the diagnostic value of plasma HSP90α in HCC patients.

Compared to previous studies, the interesting findings here are that plasma HSP90α levels were associated with age, and significantly higher levels existed in those HCC patients who were less than 50 years old. The reason for the differential levels of plasma HSP90α in different age groups is still unclear, and more studies are needed to confirm this result. Moreover, the relationship between the plasma HSP90α levels and tumor size, tumor number, EHM, PVTT and Child-Pugh class were analyzed in the current study and the results demonstrated that HCC patients with EHM or PVTT or greater tumor size or multiple tumors is associated with high levels of plasma HSP90α. Therefore, we speculated that the levels of plasma HSP90α might be related to prognosis. In addition, the plasma HSP90α levels were significantly higher in those HCC patients who were at advance stages of the disease compared to those patients at an early stage, and this result is consistent with recently published studies [8, 9, 21]. Moreover, it was shown that there was an increasing trend of HSP90α levels with the progression of BCLC stage. Therefore we speculated that the levels of plasma HSP90α may play a key role in determining the disease stage. In addition, an interesting finding in the current study was that the levels of plasma HSP90α were significantly higher in the patients with AFP levels greater than or equal to 400 ng/mL compared to those patients with AFP levels of less than 400 ng/mL. However, the levels of HSP90α protein when assessed by immunohistochemistry in HCC tissue samples showed no association with the levels of serum AFP in a previous study [21]. Hence, the quantitative test of HSP90α levels in peripheral blood plasma samples was a better means of measuring the expression levels than the qualitative measurements derived from assessing the levels of HSP90α in tissue samples by immunochemical techniques.

The results of ROC curve analysis showed that by comparison with AFP (AUC 0.922, sensitivity 81.18%, specificity 93.91%, cut-off 5.38 ng/mL), the plasma HSP90α levels (AUC 0.836, sensitivity 67.07%, specificity 90.43%,cut-off 69.10 ng/mL) have a poor performance in the diagnosis of HCC from healthy donors. In addition, a further subgroup analysis showed that the plasma HSP90α have a limited diagnosis efficiency for early HCC patients with tumors of less than 2 cm or those at an early BCLC stage (ie. BCLC-A). However, this result is inconsistent with the recent study that showed plasma HSP90α (AUC 0.965, sensitivity 93.3%, specificity 90.3%) improved significantly compared with AFP (AUC 0.887, sensitivity 61.1%, specificity 96.3%) for the diagnostic ability of distinguishing HCC or early-HCC from non-liver cancer control patients [9].

However, from another study, we found that the diagnostic efficiency of AFP for HCC patients was AUC 0.67 with a sensitivity of 47.8% and a specificity of 93.2% [22]. A possible reason for this discrepancy of ROC curve results could be the difference of control subjects in the two studies. Even so, the combination of HSP90α and AFP significantly improved the diagnostic efficiency for HCC compared to the use of a single marker in both, the current study (AUC 0.943, sensitivity 85.89%, specificity 98.26%) and the previous study (AUC 0.977, sensitivity 93.7%, specificity 94.4%) [9]. Here we can see that the AFP levels have a high specificity for the diagnosis of HCC, but this is coupled with poor sensitivity. Considering that the use of a single protein as a biomarker has the limitations of both sensitivity and specificity, the combination of HSP90α, AFP and potentially another clinical index or biomarker might improve the diagnostic efficiency and staging determination for HCC in the future.

## Conclusions

The levels of plasma HSP90α in healthy donors, benign liver tumor cohort, SHC cohort and HCC cohort showed a statistically significant increasing trend in this study. The levels were associated with age, BCLC stage, levels of AFP, tumor size, tumor number, EHM, PVTT and Child-Pugh class in the HCC cohort. The combination of HSP90α and AFP significantly improved the diagnostic efficiency for HCC patients and these simple tests can be useful in both rural and cosmopolitan settings. These simple and easy tests may be beneficial to patients in a rural setting where elaborate equipment is not always available.

## Supplementary information

**Additional file 1:** The raw data of healthy donors cohort.

**Additional file 2:** The raw data of hepatic hemangionma patients cohort.

**Additional file 3:** The raw data of secondary hepatic carcinoma patients cohort.

**Additional file 4:** The raw data of hepatocellular carcinoma patients cohort.

## Data Availability

All data generated or analysed during this study are included in this published article and its supplementary information files.

## Abbreviations

AFP: Alpha-fetoprotein
BCLC: Barcelona Clinical Liver Cancer
EHM: Extrahepatic metastasis
HBV: Hepatitis B virus
HCC: Hepatocellular carcinoma patients
HD: Healthy donors
HH: Hepatic hemangioma patients
PVTT: Portal vein tumor thrombus
SHC: Secondary hepatic carcinoma patients
